# Association between abnormal serum free light chains ratio and known prognostic factors in lymphoma; a nephrology viewpoint

**DOI:** 10.15171/jrip.2017.29

**Published:** 2016-12-15

**Authors:** Minoosh Moghimi, Mahshid Kashkooli Behroozi, Mehdi Maghbooli, Sattar Jafari, Saeideh Mazloomzadeh, Aiyoub Pezeshgi

**Affiliations:** ^1^Department of Hematology-Oncology, Faculty of Medicine, Zanjan University of Medical Sciences, Zanjan, Iran; ^2^Metabolic Diseases Research Center, Zanjan University of Medical Sciences, Zanjan, Iran; ^3^Department of Oncology, Shahid-Beheshti University of Medical Sciences, Tehran, Iran; ^4^Department of Neurology, Zanjan University of Medical Sciences, Zanjan, Iran; ^5^Department of Epidemiology, Zanjan University of Medical Sciences, Zanjan, Iran

**Keywords:** Lymphoma, Free light chains, Prognosis

## Abstract

**Introduction:** The serum immunoglobulin free light chain (FLC) assay quantities of free kappa (κ) and lambda (λ) light chains. This assay has prognostic value in plasma cell proliferative disorders. There are limited data on serum FLC in B-cell malignancies.

**Objectives:** The aim of this study was to compare the known prognostic factors in non-Hodgkin’s lymphoma of the type B-cell and Hodgkin disease with an abnormal secretion amount of light chains in these lymphomas.

**Patients and Methods:** The association of pretreatment FLC and FLC ratio (κ:λ) with previously known prognostic factors for lymphoma such as the international prognostic index (IPI) and B symptoms were evaluated in 50 patients with Hodgkin’s and non-Hodgkin’s lymphoma. IPI is a prognostic score given based on the clinical variables including age, disease stage, serum LDH and extra-nodal involvement. Elevated FLC and an abnormal κ:λ ratio was defined based on the previous publications.

**Results:** The prevalence of abnormal FLC ratio was 38% in all patients and 40.9% in patients with diffuse large B-cell lymphoma. Abnormal FLC ratio was significantly associated with IPI (*P*=0.04) and B symptoms (*P*=0.02) in both groups of the patients with Hodgkin’s and non-Hodgkin’s lymphoma. The stage of the disease in Hodgkin’s lymphoma patients showed a significant relationship with the abnormal FLC ratio (*P*=0.04). Presence of the B symptoms in patients with Hodgkin’s lymphoma had a modest but not statistically significant association with the abnormal FLC ratio (*P*=0.07).

**Conclusion:** Abnormal FLC ratio as a new potent prognostic biomarker has a significant association with IPI which is the most common clinical tool used to predict outcome in lymphoma patients. Since there is a need for developing a reliable and quantitative prognostic biomarker for lymphoma, evaluation of the independent effect of the abnormal serum FLC ratio is suggested to be considered in future prospective studies. The result of these studies will also be useful for nephrologists, while serum immunoglobulin FLC is capable to damage kidney.

**Implication for health policy/practice/research/medical education:**

The findings of the current study indicated that kappa to lambda ratio may be useful as a prognostic factor for B cell and Hodgkin’s lymphoma patients. However confirming this relationship in Hodgkin’s and other B cell lymphomas needs more prospective studies with more participants involved. The result of these studies will also be useful for nephrologists, while serum immunoglobulin FLC is capable to damage kidney.

## Introduction

Hodgkin’s and non-Hodgkin’s lymphomas are malignant cancer types which originate from lymphatic tissues (1). US Cancer Institute estimates the prevalence of non-Hodgkin’s and Hodgkin’s lymphomas to be 20.5 and 3 in every 100 000 people, respectively (2). Lymphoma is a cancer type with unknown risk factors and etiology. However some studies have addressed many risk factors, although their mechanisms of effect and their exact relationship remain unclear (3,4). Some of the chief risk factors for non-Hodgkin’s lymphoma are; the age being over 60 years, being male, being exposed to harmful chemicals and radiation and immunosuppression (due to HIV infection, organ transplant and other infections). The main risk factors for Hodgkin’s lymphoma are Epstein-Barr viral infection, inheritance, and infections due to AIDS (1,5,6). Lymphomas may cause various symptoms. Their most common symptom is adenopathy, especially in the neck area, and groins which is usually painless (7,8).

Various treatment protocols have been advised for lymphoma. Although studies have been unable to evaluate their exact success rate, the results show their overall success in treating lymphoma. The 5-year survival rate in 66% of patients suffering from non-Hodgkin’s lymphoma and 85% of patients suffering from Hodgkin’s lymphoma and those receiving treatment (1,9-11). For the time being, the current treatments include chemotherapy, immunological treatments using monoclonal antibodies and other biological medications, radiotherapy, bone marrow transplant and in some cases, surgery (10-13). In lymphoma, as in other diseases, investigating the patients’ prognosis is vital for the success of the treatment, helping to choose the right treatment method and, if necessary, to switch to another treatment method. Generally, prognosis varies in non-Hodgkin’s lymphoma based on histology of lymphoma stage at the time of diagnosis and the treatment response of the disease. Currently the scoring system used to prognosticate patients suffering from non-Hodgkin’s lymphoma is the international prognostic index (IPI) (14). In patients suffering from Hodgkin’s lymphoma, international prognostic score (IPS) is used to prognosticate the patients at the time of the diagnosis (15). In both of these tools a series of risk factors (five in non-Hodgkin’s and seven in Hodgkin’s lymphoma) have been included with one point being dedicated to each one of them. Therefore, patients having no or only one risk factor are considered to have good prognosis; patients having 2 or 3 risk factors are mediocre; and patients having 4 or more are assumed to have poor prognosis (14,15). Currently, these two essential tools are used for prognosticating patients. Some studies stated other elements as factors to determine patients’ prognosis. One of these factors is the amount of serum light chains, generated by lymphocyte B cells (16-18). Lymphocyte B cells are able to secrete many types of immunoglobulins either in their entirety or in their partial forms of light and heavy chains (19). Every serum light chain contains 220 amino acids and the average daily production amount of serum light chains in a normal individual is supposed to be 500 mg/d. Light chains are able to damage to the tubular cells and consequently interstitial area. The kappa chain is produced two times more than lambda chain (19,20). Recent studies on patients suffering from chronic lymphocytic leukemia (CLL) and non-Hodgkin’s lymphomas have shown a significant disequilibrium in the synthesis of heavy and light chains in patients, which leads to secretion of great amounts of serum light chains compared to heavy chains and whole immunoglobulins (20-22). Additionally in new treatments such as immunotherapy which have been developed to treat different lymphomas, this feature of lymphomas has been targeted. Hence, it seems that addressing new prognostic factors which have higher sensitivity and less cost to be vital (19,20). In several studies the accompaniment of abnormal amount of kappa and lambda serum light chains with poorer prognosis in some lymphomas has been shown to exist and this may display that the abnormal quantity of serum light chains has the potential to be accounted as an independent prognostic factor in lymphoma.

## Objectives

The aim of this study was to compare the known prognostic factors in non-Hodgkin’s lymphoma of the type B-cell and Hodgkin’s with an abnormal secretion amount of light chains in these lymphomas.

## Patients and Methods

### Study population

In the current descriptive and analytical study, 50 patients suffering from Hodgkin’s and non-Hodgkin’s B-cell lymphomas were studied. They were diagnosed using a reliable pathological sample. All the patients suffering from Hodgkin’s and non-Hodgkin’s B-cell lymphoma that were not previously treated (chemotherapy or radiotherapy), were recruited to take part and remain in the study. Those patients younger than 15, pregnant women, patients suffering from kidney failure and liver cirrhosis were exempted from the study. The sample size was determined with regards to the previous studies and for 50 participants, we assumed: α = 0.05, δ = 1.5 and d = 0.4.

After choosing the participants, a bone marrow sample was acquired from the patients except for those who were stage 1 Hodgkin. The bone marrow and blood sample were sent to a laboratory instantly to count cells, measuring beta-2 microglobulin, albumin and LDH levels. According to these data, CT-scan carried out before starting the treatment. Then patients were staged on the basis of Ann Arbor Table. On the basis of IPI and IPS scores, and based on their type of lymphoma being non-Hodgkin’s or Hodgkin’s lymphoma, the prognosis for every patient was calculated and recorded.

To investigate the serum light chains, a venous blood sample of 3 cc, without the fasting condition, was accumulated and sent to laboratory instantly and after being centrifuged and the separation of their plasma their serum light chains amount was measured using enzyme-linked immunosorbent assay (ELISA). Using this method, which is based on measuring the colored complexes of anti-gene and anti-body, the experimental samples were mixed with an unknown amount of antigen in solid phase. Then anti-body was added in order to react with the antigen and form a compound. The experimental plate was rinsed in the intervals between the stages. Before the last stage, the plate was cultivated by adding an enzyme sub-layer which led to the formation of a colored substance. The wavelength of the acquired substance color was assessed and recorded by a spectrophotometer. This wavelength indicated the presence of an antibody or antigen and its density. In this study a BioVender kit was used. Its sensitivity for kappa light chain and light lambda chain was 6 μg/L and 5 μg/L, respectively. The test accuracy for kappa light chain was intra-assay; CV = 2%-4% and inter-assay; CV = 6.9%-7% and for lambda light chain was intra-assay; CV = 3%-6% and inter-assay; CV = 5.9%-6.8%. As in the instruction of the kit, the normal level for kappa chain is 11-12 mg/L and for lambda chain 16-18 mg/L. The normal kappa to lambda ratio is considered to be 0.37-0.96. A ratio of higher than 0.96 indicates a high amount of kappa light chain and a ratio of lower than 0.37 indicates a higher than normal amount of lambda being produced.

The demographic information of the patients including age, gender, lymphoma type and their subgroup, tumor stage, B symptoms(systemic symptoms consisting of fever, weight loss and night sweats, which can be accompanied with both Hodgkin’s lymphoma and non-Hodgkin’s lymphoma) was acquired form Ann Arbor Table and bone marrow involvement.

### Ethical issues

1) The research followed the tenets of the Declaration of Helsinki; 2) written informed consent was obtained, and they were free to leave the study at any time; and 3) the research was approved by the ethical committee of Zanjan University of Medical Sciences.

### Statistical analysis

For data analysis, we applied the SPSS version 15 software. Descriptive statistics for demographic and laboratory indices was applied. Chi-square test for correlation was used. The significance level was the *P* value of below 0.05.

## Results

Table 1 illustrates the data of patients. Around 25 (50%) were female. Around, 22 (44%) suffered from Hodgkin’s lymphoma and 28 (56%) suffered from non-Hodgkin’s lymphoma. Out of 28 non-Hodgkin’s lymphoma, 22 (78%) suffered from diffuse large B-cell lymphoma, 4 suffered from small lymphocytic lymphoma (SLL) (14.5 %) and the proportion of patients suffering from hairy cell leukemia and follicular lymphoma was one for each of them (3.75%). Lambda chains level in only 4 (8%) and kappa chains in only 9 (18%) participants was at the normal level. Table 1 shows the details of age, gender, tumor level, LDH, bone marrow involvement, the results of IPS and IPI tools, the measured level of lambda and kappa chains and their relative ratio for both of the lymphoma types.

**Table 1:** Distribution of variables used in the study

**Variable**	**Number**	**Percent**
Age		
<60	36	72
≥60	14	28
Gender		
Female	25	50
Male	25	50
Type of lymphoma		
Hodgkin	22	44
Non-Hodgkin	28	56
Stage		
1,2	17	34
3,4	33	66
LDH		
Normal	16	32
Abnormal	34	68
Bone marrow involvement		
Positive	20	40
Negative	30	60
B signs		
Positive	24	48
Negative	26	52
IPI/IPS		
Good	15	30
Middle	25	50
Bad	10	20
Kappa		
Normal	9	18
Upper	41	82
Lambda		
Normal	4	8
Upper	46	92
Kappa/lambda ratio		
Normal	31	62
Abnormal	19	38

In this study, we found no significant relationship between the lambda serum light chain level and the studied variables. This result was also the same for kappa chain level (*P* > 0.05. There was no significant relationship between abnormal ratio of kappa to lambda serum light chains with age, gender, lymphoma type, lymphoma stage, serum LDH level and bone marrow involvement (*P* > 0.5). However, a statistically significant relationship between the abnormal ratio of kappa chain to lambda with B symptoms (*P* = 0.02) and IPI/IPS ratio (*P* = 0.04) was detected, which is used as a prognosis predictor of Hodgkin’s and non-Hodgkin’s patients. Likewise, in patients suffering from diffuse large B-cell lymphoma, no significant relationship between variables and light chains and their ratio was detected (*P* > 0.5).

## Discussion

This study was a descriptive study with the aim of specifying the normal alteration in the proportion of serum light chains with known prognostic factors. There has been various prognostic criteria for different lymphoma types. In these criteria, many factors such as bone marrow involvement, lymph node involvement level, serum LDH levels and age of patients at the time of diagnosis and also serum albumin level have been described (22,23). In a variety of non-Hodgkin’s and Hodgkin’s lymphomas, the primary lineage are lymphocyte B cells which are able to secrete many types of immunoglobulins entirety or partially of immunoglobulins as light and heavy chains. The abnormal levels of serum light chains have long been used as a determinant prognostic factor for plasma cell malignancies, while they had toxic effects on kidney too. Recently some studies address the helpfulness of measuring these chains in other B cell malignancies. In this study, a higher than normal value of kappa light chain level in 82% of lymphoma patients, and in 92% of them, elevated level of lambda light chain was detected. In 28% of our patients, the kappa to lambda ratio was above the normal range. In 22 patients suffering from diffuse large B-cell lymphoma, 86% kappa chain and 95.5% lambda chain levels were higher than normal threshold. Also 40.9% of the patients were reported to have an abnormal kappa to lambda ratio. In the previous studies relationship between serum light chains and Hodgkin’s lymphoma had not been investigated yet. In Hodgkin’s lymphoma patients, we found, 77.3% had abnormal kappa levels and 86.4% had abnormal lambda levels and in 27.3% of subjects the kappa to lambda ratio was abnormal. Additionally, we found that the relationship between lymphoma stage and kappa to lambda ratio was statistically significant (*P* = 0.04).

In a study by Dingli et al, the abnormal level of serum light chain was reported to be 29%. This level is significantly lower than that of the current study. It should be noted that, in their study the Hodgkin’s patients did not participate (24). In a study by Martin et al, the abnormal kappa to lambda ratio in diffuse large B-cell lymphoma was reported to be 8% (25). In another study by Maurer et al on patients suffering from diffuse large B-cell lymphoma, abnormal serum light chains and the abnormal kappa to lambda ratio was reported to be 34%, 12% (26).

In our study, no statistically significant relationship between the studied variables such as age, gender and illness stage with B symptoms and with bone marrow involvement was seen. There was not significant association of IPI with abnormal level of kappa and lambda chains and kappa to lambda ratios in patients suffering from diffuse large cells too. However in the study of Murer et al only the relationship between age with abnormal kappa and lambda chains was statistically significant (*P* < 0.001) (26). However, in the study conducted by the Maurer et al, relationship of other mentioned variables with abnormal serum light chains and the kappa to lambda ratios was not statistically significant. Importantly, despite the significant relationship between the participants’ ages and the abnormal levels of serum light chains in the study of Maurer et al, no statistically significant relationship of age with the kappa to lambda ratio was detected (26).

Accordingly, we found significant relationships of IPI/IPS and B symptoms (*P* = 0.02) with the abnormal kappa to lambda ratio level (*P* = 0.04 and *P* = 0.04 respectively), and this was not reported in previous studies.

## Conclusion

The findings of the current study indicated that kappa to lambda ratio may be useful as a prognostic factor for B cell and Hodgkin’s lymphoma patients. However confirming this relationship in Hodgkin’s and other B cell lymphomas needs more prospective studies with more participants involved.

## Limitations of the study

One of the most important limitations in this study, was the low number of lymphoma patients in Zanjan and the high cost of lambda and kappa kits. Due to the unavailability of beta-2 microglobulin kits in the hospital and the inability of transferring the samples to laboratories other than that of the hospital, this variable was not measured in the patients.‏ Future studies should be carried out with more participants and also the source of light monoclonal or polyclonal light chains in lymphoma patients and their relationship with patient prognosis should be studied.

## Authors’ contribution

All authors of the manuscript participated equally in conducting the research and preparing of the article.

## Conflicts of interest

The authors declared no competing interests.

## Ethical considerations

Ethical issues (including plagiarism, data fabrication, double publication) have been completely observed by the authors.

## Funding/Support

This study was extracted from internal medicine residential thesis of Mahshid Kashkooli Behroozi (Thesis #A-12-482-9), in Zanjan University of Medical Sciences, Zanjan, Iran.
